# Epigenetic Control of Interferon-Gamma Expression in CD8 T Cells

**DOI:** 10.1155/2015/849573

**Published:** 2015-04-20

**Authors:** Patrícia S. de Araújo-Souza, Steffi C. H. Hanschke, João P. B. Viola

**Affiliations:** ^1^Program of Cellular Biology, Brazilian National Cancer Institute (INCA), Rua André Cavalcanti 37, Centro, 20231-050 Rio de Janeiro, RJ, Brazil; ^2^Department of Immunobiology, Biology Institute, Fluminense Federal University (UFF), Outeiro São João Batista s/n, Centro, 24020-141 Niterói, RJ, Brazil

## Abstract

Interferon- (IFN-) *γ* is an essential cytokine for immunity against intracellular pathogens and cancer. IFN-*γ* expression by CD4 T lymphocytes is observed only after T helper (Th) 1 differentiation and there are several studies about the molecular mechanisms that control Ifng expression in these cells. However, naïve CD8 T lymphocytes do not produce large amounts of IFN-*γ*, but after TCR stimulation there is a progressive acquisition of IFN-*γ* expression during differentiation into cytotoxic T lymphocytes (CTL) and memory cells, which are capable of producing high levels of this cytokine. Differential gene expression can be regulated from the selective action of transcriptional factors and also from epigenetic mechanisms, such as DNA CpG methylation or posttranslational histone modifications. Recently it has been recognized that epigenetic modification is an integral part of CD8 lymphocyte differentiation. This review will focus on the chromatin status of Ifng promoter in CD8 T cells and possible influences of epigenetic modifications in Ifng gene and conserved noncoding sequences (CNSs) in regulation of IFN-*γ* production by CD8 T lymphocytes.

## 1. Interferon- (IFN-) *γ*

Interferon- (IFN-) *γ* is an essential cytokine for immunity against intracellular pathogens and cancer. This is notably clear when genetically modified animals lacking IFN-*γ* responsiveness are analyzed. Mice with targeted disruptions of the Ifng gene or Ifng gene receptor 1 are highly susceptible to a variety of bacteria, protozoans, and virus infection [1]. Furthermore, when mice lacking sensitivity to IFN-*γ* were challenged with chemical carcinogens, they developed tumor more rapidly and with higher frequency than wild type animals [2, 3].

IFN-*γ* is produced by cells that mediate both innate and adaptative immune responses. Natural killer (NK) and natural killer T (NKT) cells are the innate cells sources of this cytokine and rapidly produce IFN-*γ* upon activation. On the other hand significant increase in IFN-*γ* expression by CD4 T lymphocytes is observed only after T helper (Th) 1 differentiation. In fact, upon activation, CD4 T cells can differentiate into several effector lineages, of which Th1 is the only one that produce high levels of IFN-*γ*. Naïve CD8 T lymphocytes do not produce large amounts of IFN-*γ*, but after TCR stimulation these cells undergo differentiation into cytotoxic T lymphocytes (CTL) and memory cells, which are capable of producing high levels of this cytokine in response to TCR activation or Interleukin- (IL-) 12 and IL-18 [4]. The progressive acquisition of IFN-*γ* expression by CTL depends on continued lymphocyte proliferation [5].

The best characterized role of IFN-*γ* in CD8 T cell immunity is in enhancing class I antigen presentation pathway, which facilitates cytotoxic T cells to recognize infected cells. IFN-*γ* signaling upregulation leads to expression of MHC class I and the TAP transporter, as well as chaperones such as tapasin. IFN-*γ* also induces a replacement of the constitutive proteasome subunits, *β*_1_, *β*_2_ and *β*_5_, for the immunoproteasome subunits LMP2, MECL-1, and LMP7, an essential feature for increasing the quantity and repertoire of peptides presented in the context of class I MHC (reviewed in [1]).

IFN-*γ* also plays an important role in CD8 T cell homeostasis that is independent from its function in clearance of infection. Several studies have suggested that IFN-*γ* is a key determinant of immunodominance [6–8]. Badovinac and colleagues [6] have shown that IFN-*γ* deficient mice infected with an attenuated* Listeria monocytogenes* strain exhibited an altered immunodominance hierarchy due to an increased expansion of CD8 T cells specific for a subdominant epitope of* L. monocytogenes*. Furthermore, IFN-*γ* seems to be required for subdominant CD8 T cells response suppression by dominant CD8 T cell response [7] and the CD8 T cells that promptly produce IFN-*γ* after stimulation are preferentially expanded [8].

Another effect of IFN-*γ* has been recently described by Reis and colleagues [9]. This cytokine is important for the differentiation of TCR*αβ* ^+^CD4^+^CD8*αα* ^+^ intraepithelial lymphocytes (IELs) in the gut. IELs are lymphocytes considered “activated yet resting” and their regulation is crucial role in the maintenance of the epithelial cell barrier and gut physiological inflammation [9]. IFN-*γ* also acts directly on CD8 T cells by stimulating their abundance in an acute lymphocytic choriomeningitis virus (LCMV) infection [10] and enhancing the development of memory cells [11]. Interestingly, Sercan and colleagues [12] show that IFN-*γ* produced by innate immune cells contributes to antigen-specific CD8 T cell homeostasis. They show that IFN-*γ* directly promotes CD8 T cells expansion. However, Badovinac and colleagues [6] also have shown that IFN-*γ* deficiency resulted in a delayed contraction of antigen-specific CD8 T cell populations from both* Listeria monocytogenes* and LCMV infections, which suggests an important role of this cytokine in control of death phase of antigen-specific CD8 T cells. Therefore, this cytokine has both positive and negative effects on CD8 T cell abundance depending on the phase of the CD8 response and also the biology model evaluated. This dual role is clearly evident in IFN-*γ* deficient mice, in which both the expansion and contraction of CD8 T cell response are impaired [6].

## 2. IFN-*γ* Production by CD8 T Lymphocytes

CD8 T cells are usually characterized by their cytolytic activities involving perforin or Fas mechanisms to kill targeted cells. However cytokine secretion by CD8 T cells also has an important role in the control of intracellular infections. In 1990, Fong and Mosmann [13] suggested that Th1 cells and CD8 T cells could share cytokine mediated functions, like combating intracellular pathogens and tumors cells. They observed that alloreactive murine CD8 T cell clones produced both mRNA and protein profile characteristic of Th1 clones, which include high levels of IFN-*γ*.

It is well known that the clearance of several infections also depends on noncytolytic functions of CD8 T cells. The control of* M. tuberculosis* infections in mice requires the ability of the CD8 T cells to produce IFN-*γ* [14]. Furthermore, IFN-*γ* produced by CD8 T cells is essential to clear numerous viral infections such as measles virus, herpes simplex virus type 1, LCMV, and borna disease virus. This IFN-*γ* mediated response seems to be important to avoid tissue damage and inflammation, which is normally observed in cytotoxic CD8 T cell response (reviewed in [15]). Indeed, HIV control is associated with polyfunctional CD8 T cells, that is, epitope-specific cells expressing several effector functions, which includes the expression of cytokines such as TNF and IFN-*γ* [16].

The IFN-*γ* produced by CD8 T cells seems to have other immunomodulatory roles, acting on CD4 T cells and B cells and also on CD8 T cells themselves. The differentiation of CD4 T cells in T helper subsets depends on the cytokine milieu where these primary T cells were stimulated. The main sources of these cytokines are activated innate immune cells. For Th1 differentiation, the cytokines IL-12, produced by APCs, and IFN-*γ*, mainly produced by NK cells, are critical to induce and reinforce Th1 commitment [17].

Several studies have also suggested that CD8 T lymphocytes could have a role in the generation of Th1 immunity and in the inhibition of Th2 response [18–20]. Uzonna and colleagues [20] have shown that IFN-*γ* produced by murine CD8 T cells, in response to low doses of* L. major*, downregulates an initial Th2 response and enhances Th1 commitment. Data from our group suggest that, besides NK and dendritic cells, CD8 T lymphocytes are also another source of IFN-*γ* that enhances CD4 Th1 phenotype development [21]. We have shown that, after TCR activation of primary lymph node cells, CD8 T lymphocytes are the major source of IFN-*γ* production. Furthermore, CD4 T cells cocultured with IFN-*γ*-competent CD8 T cells clearly produce more IFN-*γ* and less IL-4 than CD4 T cells cultured with IFN-*γ*-deficient CD8 T cells. This work also suggested that NFAT1 transcription factor-dependent IFN-*γ* production by CD8 T cell is important during eosinophil migration to pleura in a pleurisy model, which suggests an important role for IFN-*γ* produced by CD8 T cells in the control of allergic diseases. Together these studies reinforce the role of IFN-*γ* produced by CD8 T cells in regulation of Th immune responses* in vivo*.

The autocrine IFN-*γ* signaling is important for Th1 differentiation, and recently it has been suggested that IFN-*γ* is an autocrine/paracrine factor to promote naïve T CD8 cell differentiation [22]. Low levels of IFN-*γ* produced by CD8 T cells promote the upregulation of T-bet, granzyme B, and IFN-*γ* also promotes a week cytolytic activity. IFN-*γ* alone does not support strong differentiation but can synergize with IFN-*α* in driving effector differentiation of these CD8 T cells. A previous paper has also shown that naïve CD8 T cells receive an IFN-*γ* signal few hours after* L. monocytogenes* infection [23]. These studies suggest that the IFN-*γ* signals for CD8 T cell differentiation are delivered early in the immune response.

When naïve T CD4 and CD8 cells are compared for the IFN-*γ* production, it is clear that, after TCR activation, CD8 T cells produce more levels of IFN-*γ* than CD4 T cells [21], and the requirements for TCR induced IFN-*γ* production are different between these primary cells [24]. In fact, Carter and Murphy [24] have shown that CD4 T cells require STAT4 activation beside TCR signalization to produce IFN-*γ*, while CD8 T cells need only TCR activation. Furthermore, the main difference in transcriptional requirements for Ifng expression between CD4 and CD8 T cells seems to be the differential role of members of the T-box family of transcription factors, T-bet and Eomesodermin (Eomes). T-bet is the master regulator of Ifng and Th1 commitment of CD4 T cells [25] and sufficient for induction of Ifng expression in these cells. On the other hand, IFN-*γ* production by CD8 lymphocytes is also dependent of Eomes expression [26]. Additionally, Eomes and T-bet play important roles during CD8 T cell differentiation to effector and memory T cells, where T-bet is associated with effector phenotype whereas expression of Eomes increases in memory CD8 T cells [27].

There are several studies about IFN-*γ* production by Th1 cells and the molecular mechanisms that control Ifng expression in these cells are widely investigated and extensively reviewed. However, regulation of Ifng expression in CD8 T cells is not fairly explored and discussed.

## 3. Epigenetic of Ifng in CD8 T Cells

### 3.1. Ifng Promoter

An effective cellular immune response is characterized by robust stimulation of naïve lymphocytes to undergo differentiation into effector cells, which provides pathogen clearance while promoting the development of long-lived memory cells that can respond to reinfection faster than naïve cells [28]. The CD8 differentiation is accompanied by large-scale changes in the coordinate expression of genes associated with effector function, survival, and self-renewal [29] and recently it has been recognized that epigenetic modification is an integral part of this process. This section will focus on the chromatin status of Ifng promoter in CD8 T cells. However, due to the scarcity of studies exploring CD8 T cells and the higher availability of data investigating CD4 T cells, some aspects of CD4 regulation will be mentioned for comparison.

Differential gene expression can be regulated from the selective action of transcriptional factors and also from epigenetic mechanisms, such as DNA methylation or posttranslational histone modifications. These epigenetic modifications could be heritable and occur without affecting the DNA sequence, which makes the epigenetic information potentially plastic. It is the whole set of epigenetic modifications at a given locus, including the interaction of ATP-dependent nucleosomal remodeling complexes with DNA methylation and histone modifications that play a key role in regulating gene expression and chromatin organization.

Methylation of cytosine residues within CpG dinucleotides is an efficient epigenetic mechanism for gene silencing. The methyl group addition at the 5′ carbon of the pyrimidine ring of cytosine is catalyzed by enzymes called DNA methyltransferases (Dnmts). DNA methylation in the vicinity of transcriptional start sites results in repression and gene silencing by direct and indirect mechanisms. The direct mechanism is done by affecting the binding of transcription factors that do not recognize methylated CpG sites. Indirect mechanisms include the binding of several proteins to methylated cytosines. These proteins prevent the binding of transcription factors to DNA and can recruit several enzymes that catalyze transcriptionally silent histone modifications and other factors that make the chromatin more compact and consequently less accessible to transcription machinery [30].

**Figure 1:**
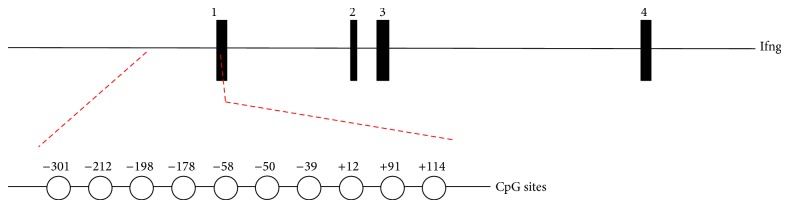
Schematic view of mouse interferon-*γ* locus (Ifng). Exons are shown as black boxes. In detail, the relative positions of the CpG sites located at the Ifng promoter are indicated. The numbers correspond to their distance relative to the transcription start site (+1) of the Ifng.

The importance of CpG methylation for Ifng expression is supported by experiments of CD8 T cell cultures stimulated via TCR in the presence of 5-azacytidine (5-AZA), a drug that causes DNA demethylation upon proliferation. Upon AZA treatment, increased levels of this cytokine in culture supernatants were described [31], as well as an increment in the number of naïve cells able to produce IFN-*γ* when compared to control [32]. Several genomic regions could be involved in this regulation and a possible role of Ifng mouse promoter was particularly investigated. The ~600 pb region contains 10 CpG sites (Figure 1). Numeration of these sites varies among different publications, but here we will denominate it according to the initial transcription site from the RefSeq sequence identifier NM_008337.

CD8 T cell clonal analysis of Ifng promoter methylation by bisulfite genomic DNA sequencing and mRNA expression by quantitative competitive PCR (QCPCR) revealed an overall association between demethylation of IFN-*γ* promoter and expression of mRNA. CpG sites located at −212, −198, and −58 were methylated in most IFN-*γ*-negative mRNA clones and their demethylation was closely related to IFN-*γ* expression [31]. However, there is a clonal heterogeneity, with clone- and site-specific differences across the promoter. Interestingly much more variability was observed when clones derived from naïve CD8^+^CD44^low^ T cells were assayed [31]. On the other hand, most of the CpG sites in Ifng promoter of clones derived from CD8^+^CD44^high^ T cells were demethylated and these clones expressed high levels of IFN-*γ*. In line with that Winders and colleagues [33] showed by Southern blot analysis with methylation-sensitive enzymes that IFN-*γ* promoter is mostly unmethylated at the −301, −212, and −58 sites in CD8 T cells from OT-I mice. Similar patterns were observed in CD4 T cells from 5C.C7 mice, and earlier stages of T cell development also revealed hypomethylation at the −212 to −50 CpG sites. But the upstream site −380 was hypermethylated in both double positive and double negative thymocytes. In accord with the hypothesis that CpG methylation occurs in IFN-*γ* nonproducers, B cells presented a completely methylated pattern. Also, stimulation of CD4 T cells in Th2 polarizing conditions leads to a pronounced increase of methylation at particular sites [33].

Kersh and colleagues [32] performed by bisulfite sequencing an* ex vivo* analysis of naïve and* in vivo* generated effector and memory CD8 T cells from P14 TCR-transgenic mice, which are specific for gp33-41 epitope of LCMV glycoprotein. Similar to that observed by Winders and colleagues [33], CD8 naïve cells presented virtually all CpG sites located between sites −212 and −39 unmethylated. But these cells have posttranscriptional sites (+12, +91, and +114) mainly methylated. Although the average numbers of methyl-CpG sites in naïve and memory cells were the same (2.8 and 2.7, resp.), methylation of IFN-*γ* promoter in memory cells is more evenly distributed. Similar to data from Northrop and colleagues [34], effector CD8 cells had a completely unmethylated promoter [32]. The discrete differences in DNA methylation between naïve and memory CD8 cells may represent a differential regulation in these cell types, because after 5 hours of antigenic-stimulation, a demethylation independent of DNA replication was observed in memory cells, but this is not true for naïve P14 cells. Furthermore, treatment of naïve P14 cells with 5-AZA led to an increase in the number of cells able to produce IFN-*γ*, and no difference was observed when memory cells were used suggesting that Ifng is silenced by DNA methylation in naïve, but not CD8, memory cells [32].

Not only the number but also the amount of intracellular IFN-*γ* produced by naïve P14 cells treated with 5-AZA has increased, when compared to control [30]. In line with that, Makar and Wilson [35] reported that when naïve CD8 T cells deficient in the maintenance Dnmt 1 (Dnmt1^−/−^) are stimulated for 3 days they increase Ifng expression 5–10-fold after restimulation. But it is important to note that even in Dnmt1 deficient mice the IFN-*γ* production is higher in CD8 than CD4 lymphocytes, suggesting that maintenance of IFN-*γ* expression in specific T cell subsets is not dependent on CpG methylation. In opposition, Th2-related cytokines (IL-4, IL-5, and IL-13) were significantly expressed only when CD8 T cells lacks Dnmt1.

The other study characterizing CpG methylation of IFN-*γ* promoter in several cell types investigated the methylation status of the −212, −198, −178, −58, −50, −39, +12, and +91 CpG sites [36]. All the CpGs were almost completely methylated in kidney and heart tissues, and entirely unmethylated in NK cells. Consistent with other reports [32, 33], CpGs located at untranscribed regions were hypomethylated and those at transcribed regions were hypermethylated in CD8 naïve cells from C57/BL6 mice. Stimulation of these cells resulted in reduction of methylation in both regions. Although a different mouse lineage was used to obtain memory CD8 T cells, they presented 0–6% and 30–70% methylation in untranscribed and transcribed region, respectively [36].

As previously reported [33], earlier stages of T cell development (DN, DP, CD4, and CD8 thymocytes) have also hypomethylated CpG sites at IFN-*γ* promoter. CD4 T lymphocytes from the same mice presented a similar CpG methylation pattern of C57/Bl6 CD8 naïve cells, but polarization to Th1 results in a significant level of demethylation in transcribed region. Like other reports, Th2 underwent some level of methylation at untranscribed region, with the position −58 being more methylated than other promoter sites, while no detectable change was observed when differentiation occurred at neutralizing conditions (Th0) [36].

Taken together, all these data suggest that naïve CD8 T cells exhibit a low methylation profile in untranscribed region and a hypermethylation in transcribed region, and, following TCR stimulation, methylation decreases in both regions.

The functional significance of IFN-*γ* promoter CpG methylation was assayed by luciferase reporter assays showing that methylation of the whole IFN-*γ* promoter vector inhibits its transcriptional activity [33, 36]. Methylation of the −212, −198, and −178 sites individually did not affect the activity of Ifng promoter, and little effect was observed when the −50 and −39 sites were exclusively methylated, but modification of −58 CpG site significantly reduced the activity to a level similar to that of the observed for the completely methylated vector [36]. Interestingly, methylation of −58 site occurred faster and more completely than the other sites during Th1 and Th2 polarization, but a more accentuated outcome was observed in Th2 [33, 36].

The versatile profile of the −58 site could potentially interfere with transcription. The use of oligonucleotide probes with methylated −58 CpG, but not −50 and −39, abolished the formation of 2 complexes verified when unmethylated probes were used in EMSA with nuclear extracts of Th1 cell line AE7. In supershift assays c-jun and ATF2 were identified in the upper band and CREB and ATF1/CREB in the lower one, and this was also observed when Th2 nuclear extracts from D10 lineage were used [36]. Chromatin immunoprecipitation (ChIP) assays confirmed the CREB and CREB/ATF1 binding and identified FosB, JunB, c-Jun, and ATF2 interacting with IFN-*γ* promoter in Th1 cell lineage* in vivo*. Concerning the Th2 cell lineage, c-Jun, ATF2, and CREB binding was not identified.

The mentioned functional and binding assays were performed in Th cells, and although they are related to CD8 lymphocytes, several data suggest that Ifng regulation may have particular characteristics in each T cell subset, like data obtained from transgenic mice model which express the luciferase gene under the control of proximal (−70 to −44) and distal (−98 to −78) regulatory elements from the IFN-*γ* promoter [37].* In vitro* primed CD4 T cells express reporters under control of both elements, while CD8 cells do so only under the distal element. In addition, elevated cyclic AMP inhibited transcriptional activity directed by the proximal regulatory element in primed CD4+ T cells but enhanced transcriptional activity directed by the distal in primed CD8+ T cells.

This differential gene expression depends on the selective action of transcriptional factors, but also from epigenetic modifications that could change the chromatin accessibility to transcriptional machinery. For example, histones can be posttranslationally modified at several amino acids residues. Depending on both covalent modification type and the modified residue, these modifications could result in gene activation or silencing. Acetylation of lysines of histones H3 or H4 (AcH3 and AcH4, resp.) and methylation of lysine 4 of histone H3 are histone modifications associated with poised or transcriptionally active genes [38]. On the other hand, trimethylation of lysines 9 or 27 of histone H3 is typically found in silenced genes [38]. In fact, reduced repressive H3K27me3 and H3K27me2 throughout Ifng after primary infection and persistence in memory CD8 T lymphocytes was reported [39], as well as a detection of minor H3K4me3 peaks near Ifng TSS in effector and memory T CD8 cells.

In addition to the loss of DNA methylation at IFN-*γ* promoter in the differentiation of naïve P14 CD8 T cells to effector, Northrop and colleagues [34] reported a more pronounced demethylation of the IFN-*γ* enhancer at the first intron. Investigation of AcH3 by ChIP and detection by real-time PCR revealed a significant increase in IFN-*γ* promoter after stimulation [40], and similar increase was observed in the comparison of naïve CD8 T cells with effector and memory CD8 T cells in promoter and enhancer [34]. Nevertheless, when CD8 memory and effector cells were generated in CD4 deficient mice (B6 CD4^−/−^), they produced “considerably” less IFN-*γ* per cell and the shift in histone acetylation is no longer seen, suggesting that demethylation of CpG sites within IFN-*γ* promoter and enhancer in CD8 effector and memory cells occurred independently of CD4 T help, while histone acetylation at these same regions was highly dependent upon the presence of CD4 help. This effect is cytokine specific, because hypoacetylation of IL-2 does not change in differentiated CD8 cells [34].

### 3.2. Regulatory CNS in Ifng Expression

Although the above data suggest that epigenetic regulation of the Ifng promoter may interfere in its expression, transgenes containing the 8.6-kb fragment of human genomic DNA containing the full length IFN-*γ* gene (promoter, introns, and up to 3.4 kb of 5′ flanking sequence) do not confer proper T cell subset-specific expression* in vivo* [41–43]. This result suggests the requirement of distal regulatory elements for suitable expression pattern. Indeed, a transgenic model containing the human IFNG gene and 90–95 kb of flanking sequence results in high-level, Th1-specific IFN-*γ* production [43]. Therefore, the search for conserved noncoding sequences (CNS) among different species has been proved as a good method for identification of relevant* cis*-regulatory elements for IFN-*γ* gene.

*In silico* searches for CNSs across human, mouse, and rat genomes allowed the identification of 2 sequences located at −5.27 kb [44, 45] and −17.36 kb of murine Ifng translational start site [44]. The first one is referred to as CNS-1 or IFN-*γ* 5′ CNS and the second, CNS-2. Both of them exhibited enhancer like function in luciferase reporter assays in response to ionomycin in T cell lineages and correspond to a DNAse I hypersensitive site in Th1 cells but not in Th2 cells [44, 45]. Similar pattern of AcH3 (and more slightly AcH4) and H3K4me2 is observed in CD8 and Th1 cells, which are good sources of IFN-*γ* [40, 44]. Interestingly, despite the fact that* in vitro* primed effector CD8 T cells have more levels of AcH3 at Intron 3 compared to CNS-1, TCR Tg effector CD8 T cells primed* in vivo* displayed greater AcH3 at CNS-1 compared to Intron 3 [40]. Increased levels of mentioned modifications were detected at promoter, Intron 3, and CNS-2 regions while Th2 has more discrete peaks. Interestingly, H3Ac ChIP analysis of Th1, Th2, and CD8 cells from mice deficient in T-bet revealed that this transcription factor is required for induction of histone modifications in Th1, but not T CD8 cells [44]. Despite some similarities between CD4 and CD8 T lymphocytes, CD8 cells seem to have particular regulation of IFN *γ* production. Therefore, more research is needed to characterize possible pathways engaged in this control.

Hatton and colleagues [46] identified a CNS located at −22 kb from Ifng that when deleted blocks Ifng reporter expression in Th1 and CD8 T cells. Transgenic mouse model also suggests that this element is required for IFN-*γ* expression in CD4 and CD8 T cells.

In 2007, Schoenborn and colleagues [47] also performed a comparative genomic analysis that revealed eight highly conserved noncoding sequences (CNSs) in a ~100 kb region surrounding Ifng. Previously identified CNS-1 (also called CNS-6), −22, −34, −54, and +18/20 (also called CNS-2) were among them, but they further identify the CNS+29, +46, and +55. Although the aim of the study was to characterize regulatory elements that govern Ifng expression in CD4 T cells, functional elements identified were also investigated in primary CD8 T cells: CNS-6 enhanced expression in stimulated CD8 cells as well as in Th0 or Th1 cells, but the authors report that CNS-34 did so only in CD8. On the other hand, CNS–22 enhanced expression in response to IL-12 plus IL-18 in Th0 and Th1 cells, but this effect was not consistently evident in CD8+ T cells.

These results suggest that some sequences like CNS-22 may be necessary for Ifng expression in both CD4 and CD8 T cells, while others, as CNS-34, may have regulatory role only in CD8 T lymphocytes. Moreover, more studies are needed to explore CNSs functions and epigenetic marks in CD8 T cells.

## 4. Concluding Remarks and Perspectives

There are relatively concordant data concerning CpG methylation of Ifng promoter, but fewer studies investigated the status of other relevant sequences for IFN-*γ* expression in CD8 lymphocytes. There is also a relative lack of data concerning other epigenetic marks, as histone modifications, and the interplay between factors that may determine or influence this status in CD8 T cells. It would be of particular interest to investigate if epigenetic events can influence heterogeneous features of CD8 T cell populations, like the capacity of polyfunctional cells to express several cytokines, and if specific microenvironments could modulate the Ifng expression through epigenetic marks, as in IELs. These observations reinforce that more studies are needed to understand the transcriptional regulation of Ifng in CD8 T cell lineage.
